# Plexiform Schwannoma of the Foot: A Case Report

**DOI:** 10.7759/cureus.84867

**Published:** 2025-05-27

**Authors:** Maryam Hammad, Kawther AlJamri

**Affiliations:** 1 Pathology, Salmaniya Medical Complex, Manama, BHR; 2 General Practice, Royal College of Surgeons in Ireland Medical University of Bahrain, Muharraq, BHR

**Keywords:** foot, neurofibroma, pathology, peripheral nerve, plexiform schwannoma, schwannoma, soft tissue

## Abstract

Plexiform schwannoma a rare subtype of schwannoma. Often presenting equally in males and females, especially in childhood. Although the causes are still unidentifiable, plexiform schwannomas are common in the head and neck regions. Hence, we present a case of plexiform schwannoma in a 14-year-old male with an uncommon location, specifically in the sole of the foot. Histopathological examination revealed features characteristic of plexiform schwannoma, including Antoni A and B areas, Verocay bodies and strong S100 positivity. The lesion was excised completely to reduce the risk of recurrence. Although these schwannomas are considered mostly benign, histological and immunohistochemical examination are necessary to rule out more sinister pathologies and guide management.

## Introduction

Plexiform schwannoma (PS) is a subtype of schwannomas accounting for 5% of schwannomas [1,2]. It typically occurs in cutaneous or subcutaneous locations and has a distinctive plexiform intraneural-nodular growth pattern [3]. PS typically presents in childhood with equal incidence in males and females in the form of an asymptomatic, slow-growing lesion measuring less than 2 cm in size [1,2]. Most plexiform schwannomas are solitary lesions and have no identifiable risk factors that have been documented in recent literature [4]. However, some associations were made with multiple PS lesions, such as a history of trauma, neurofibromatosis type-2, a relevant family history, schwannomatosis, and Gorlin-Koutlas Syndrome, to name a few [4]. PS lesions can present in multiple locations, but mostly they favor the head, neck, trunk, and upper extremities [1,2,4]. Histologically, schwannomas exhibit the following characteristics: Antoni A areas, which are densely cellular with palisading nuclei and Verocay bodies, and Antoni B areas, which are hypocellular with a myxoid matrix [5]. The best choice of treatment for PS is complete surgical resection to minimize the risk of recurrence [2,6,7]. Herein, we present a case of a PS lesion in the sole of the foot and discuss the different diagnostic and treatment options with an emphasis on histopathological findings.

## Case presentation

This is a case of a 14-year-old male with no significant past medical history, who was referred to the orthopedic clinic for an asymptomatic, stable left foot swelling on the plantar aspect between the first and second toes. His mother stated that the swelling had been present since birth and has been stable in size with no symptoms of pain or discharge. The patient also denied a history of trauma to the foot. His laboratory workup was within normal range, and there were no signs of inflammation. Physical examination revealed a soft, tender, 2 cm non-pulsatile lesion without erythema, discharge, or neurological deficits. Furthermore, his family history was negative for any significant genetic mutations.

**Figure 1:**
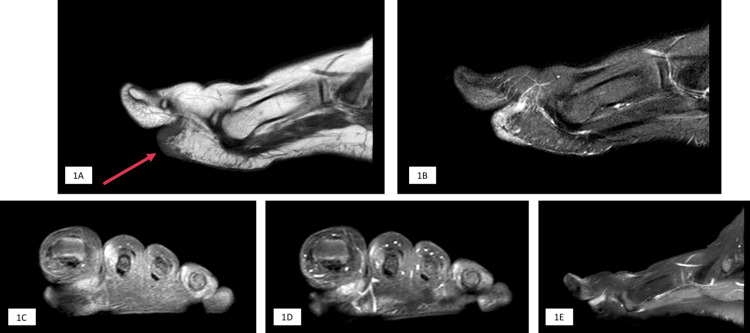
MRI sequences of the left foot (A) Sagittal T1-weighted image (B) Sagittal T2-weighted Short tau inversion recovery (STIR) image (C) Coronal T1-weighted fat-suppressed image (D) Coronal post-contrast T1-weighted fat-suppressed image (E) Sagittal post-contrast T1-weighted fat-suppressed image. Magnetic resonance images show soft tissue thickening and swelling involving dermal and subcutaneous tissue at plantar aspect of the big toe at level of proximal phalanx measure approx. 0.5 cm thickness and 2 cm length showing mild post contrast enhancement. Underlying soft tissue, tendon and bone appears intact.

Plain radiographs of the feet were requested; however, no foreign body could be seen. An ultrasound scan was performed that showed an ill-defined subcutaneous lesion, but no calcifications were noted within. A Magnetic Resonance Imaging study (MRI) was also done and reported a soft tissue swelling and thickening involving dermal and subcutaneous tissue at the plantar aspect of the big toe at the level of the proximal phalanx, measuring approximately 0.5 cm in thickness and 2 cm in length and showing mild post-contrast enhancement. The underlying soft tissue, tendon, and bone appeared intact otherwise (Figures 1A-1E).

Consequently, the treating physicians decided to schedule the patient for a complete surgical excision under general anesthesia. Surgical findings included a superficial hard soft tissue swelling located in the plantar aspect of the foot between the first and second toe. The surgery was uneventful, and the patient recovered well post-operatively. He was discharged on a follow-up basis, awaiting the histopathological result of the excised lesion.

**Figure 2:**
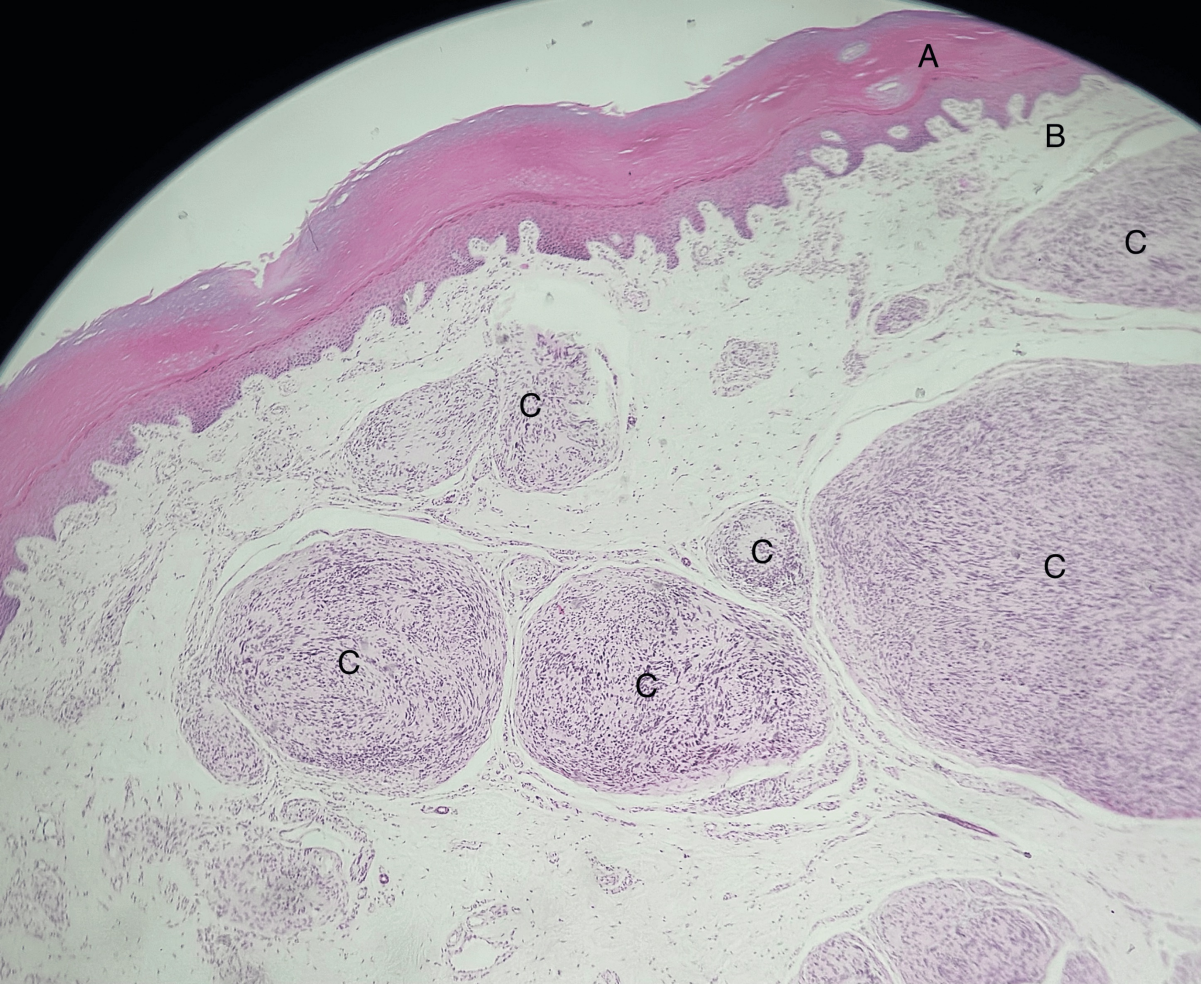
Hematoxylin and Eosin (H&E) stain at magnification power x5 (A) Epidermis (B) Dermis (C) Plexiform bundles of tumor cells. Unencapsulated, multinodular lesion composed of a proliferation of spindle-shaped cells arranged in a plexiform pattern with formation of variably sized bundles within the dermal layer extending from superficial dermis to deep cutaneous tissue.

The lesion that was sent for histopathology was further evaluated. Gross examination of the sample showed two soft tissue masses covered by skin. The larger piece measures 2 x 3 x 1.5 cm, and the smaller piece measures about 1.5 x 1 x 0.8 cm. The masses are well circumscribed with a tan-white appearance on sectioning; however, no areas of necrosis or significant hemorrhage were noted. On the other hand, microscopic examination of the sections showed skin lined by unremarkable epidermis. While Dermal layers revealed an unencapsulated, multinodular lesion composed of a proliferation of spindle-shaped cells arranged in a plexiform pattern with formation of variably sized bundles extending from superficial dermis to deep cutaneous tissue (Figure 2).

**Figure 3:**
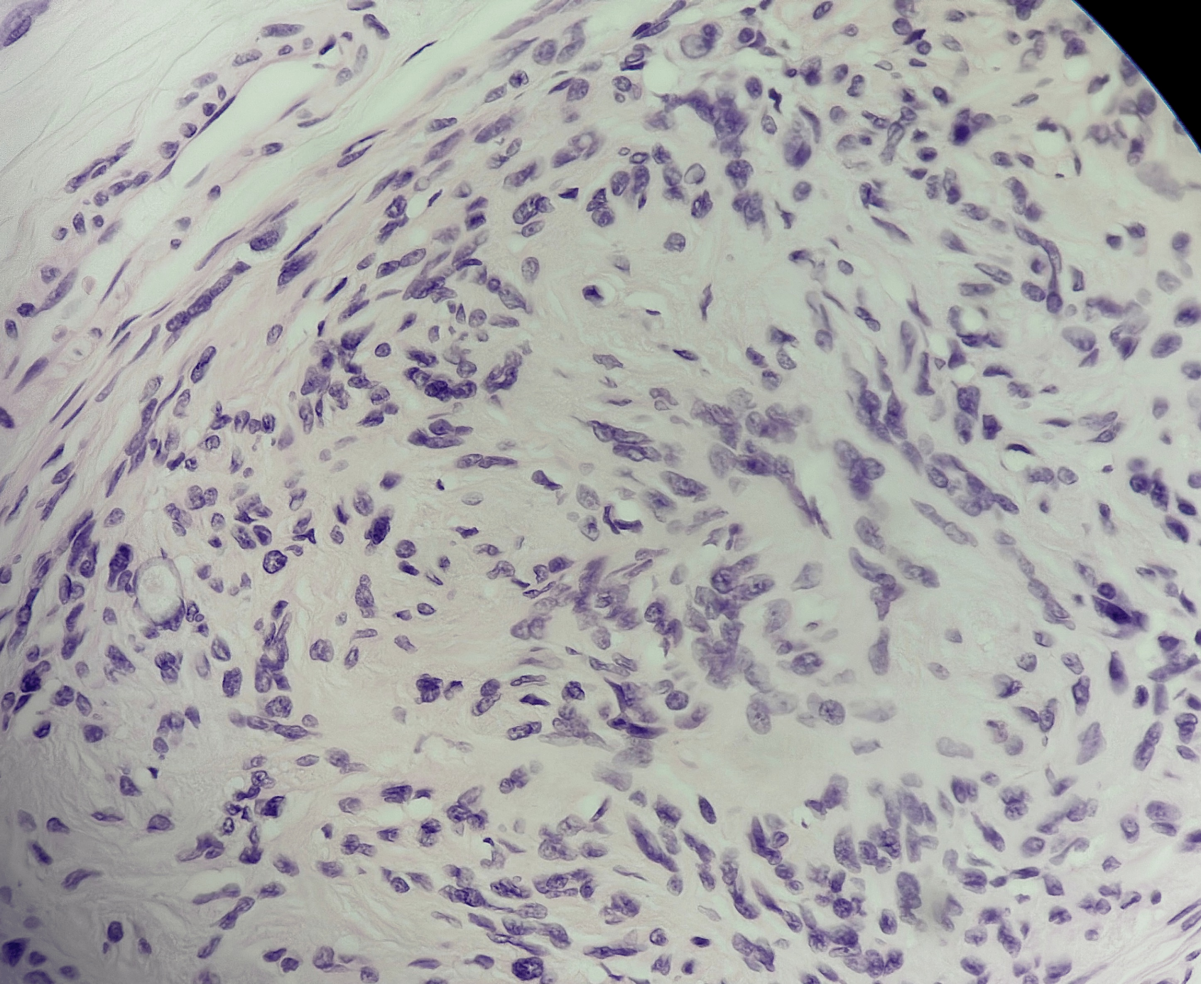
Hematoxylin and Eosin (H&E) stain at magnification power x40 Tumor cells demonstrating a characteristic wavy appearance, with areas of nuclear palisading and other areas of hyalinization.

The tumor cells demonstrated a characteristic wavy appearance, with areas of nuclear palisading and other areas of hyalinization (Figure 3).

**Figure 4:**
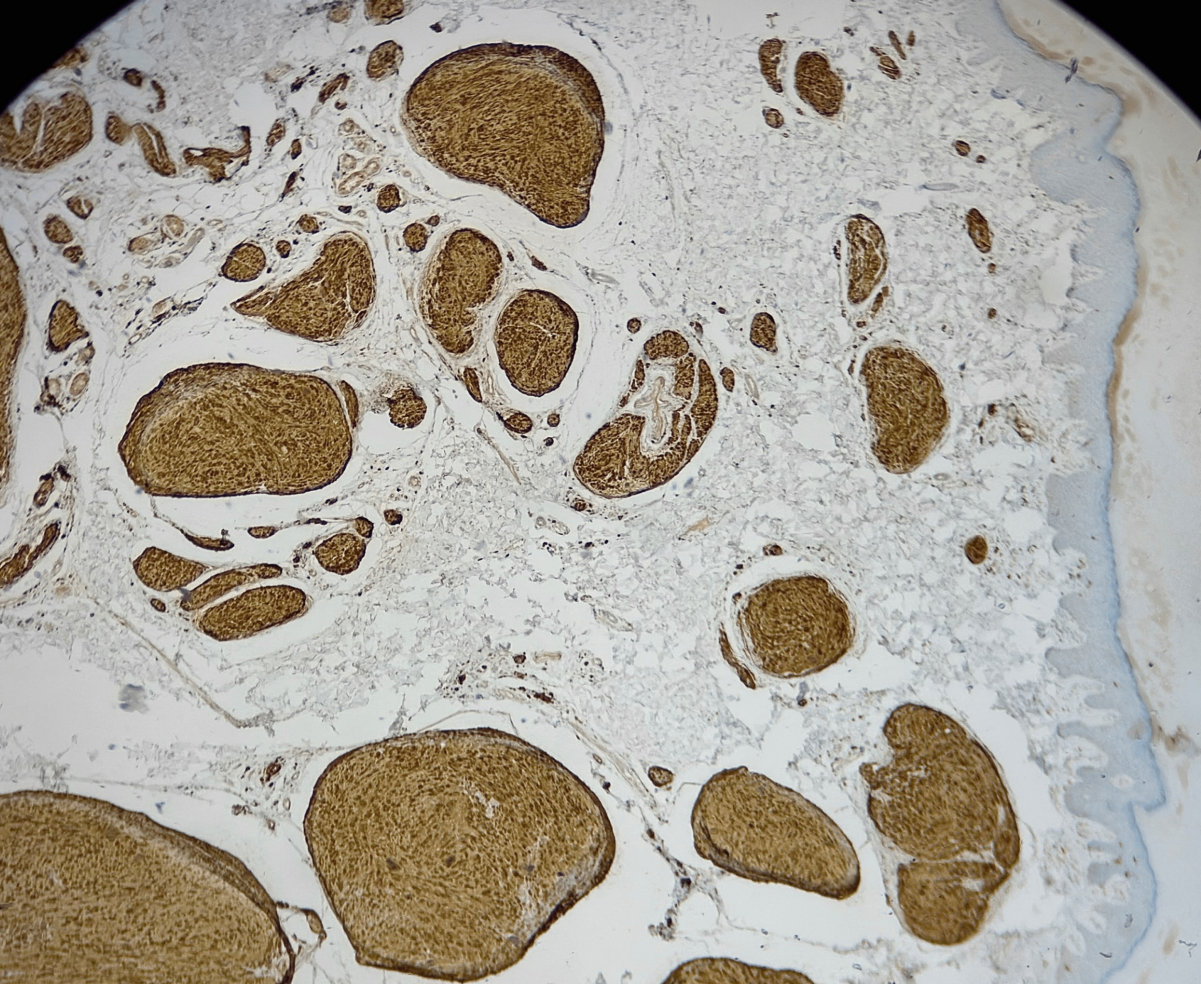
S100 immunohistochemical staining (magnification power x10) Lesional cells strongly and diffusely positive for factor S100.

**Figure 5:**
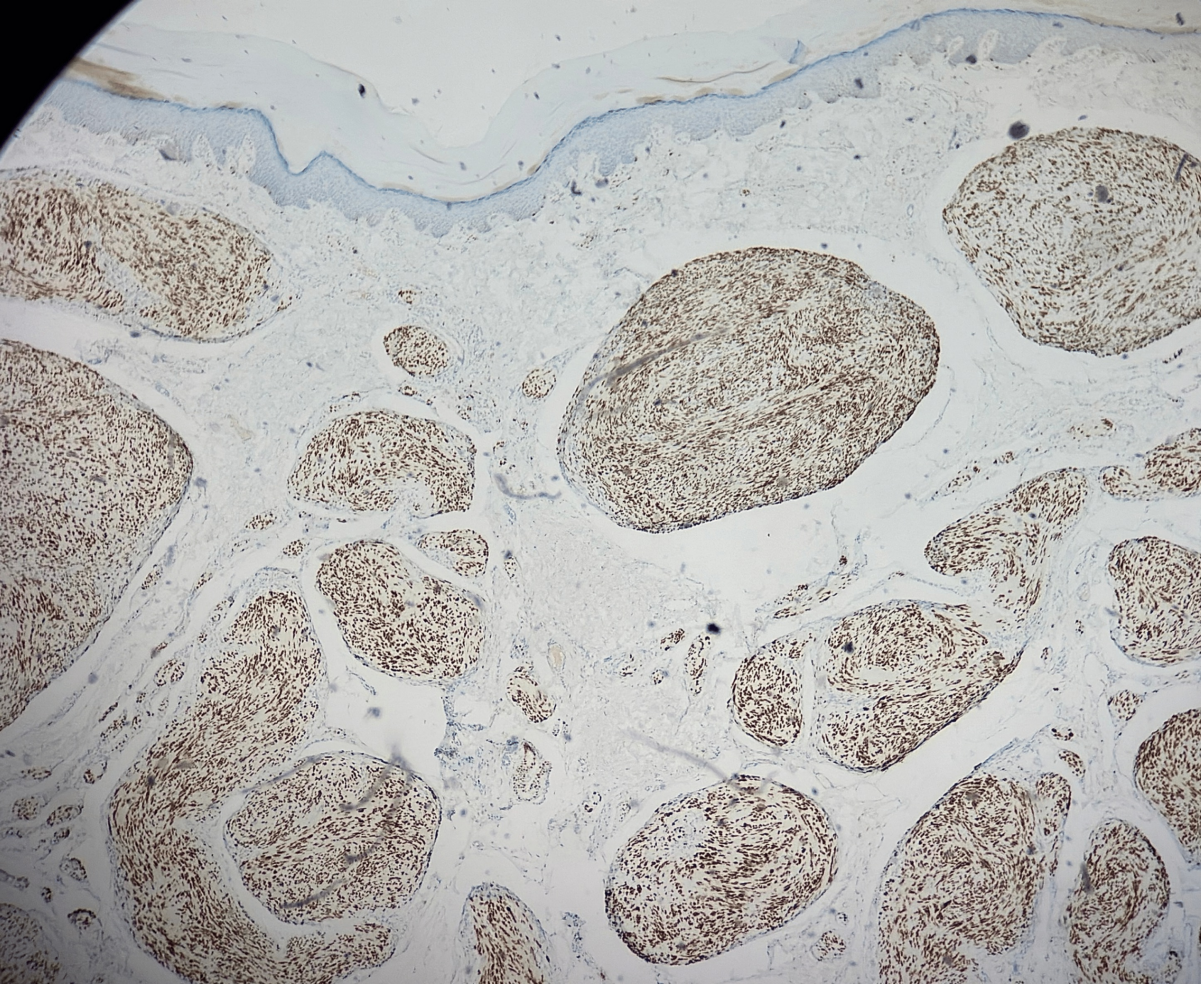
SOX10 immunohistochemical staining (magnification power x10) Lesional cells strongly and diffusely positive for factor SOX10.

No significant atypia, mitosis or necrosis were seen. The lesion appeared to be extending to the deep and lateral margins focally. Lastly, immunohistochemistry studies showed lesional cells that were strongly and diffusely positive for the factors S100 and SOX10 (Figures 4, 5). S100 and SOX10 are highly sensitive markers for Schwann cells [8].

**Figure 6:**
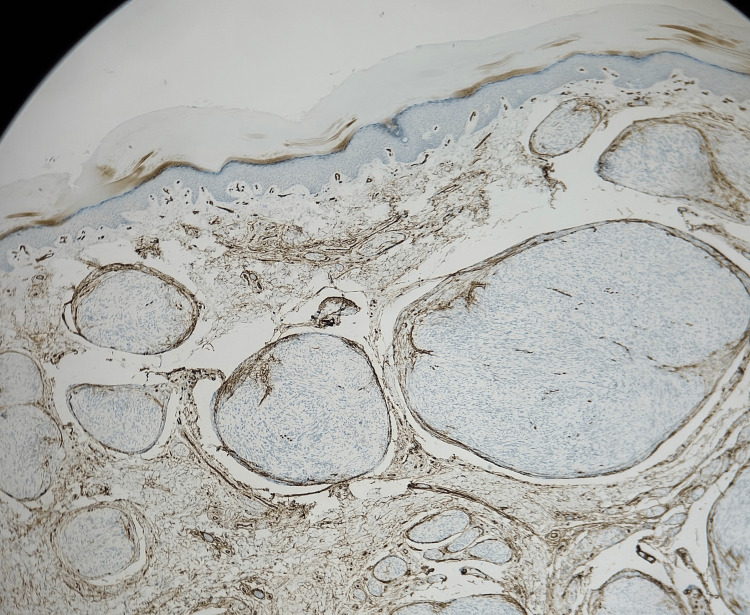
CD34 immunohistochemical staining. Magnification power x10. Lesional cells negative for CD34.

Alternatively, lesional cells were negative for CD34 (Figure 6).

The Proliferation index which was estimated by Ki67% appeared to be low. Immunohistochemical staining CD34 and Ki67% were performed to help differentiate plexiform schwannoma from other spindle cell tumors. The absence of CD34 expression and the low Ki67% helped rule out other diagnoses such as neurofibroma and malignant peripheral nerve sheath tumors [8]. Based on these findings, the final diagnosis was suggested to be a benign nerve sheath tumor consistent with a plexiform schwannoma.

## Discussion

Plexiform schwannoma (PS) is a rare subtype of peripheral nerve sheath tumors that originates from Schwann cells [1]. Plexiform schwannoma (PS) accounts for only 5% of all other subtypes of schwannomas [1]. Other subtypes of schwannoma include cellular, melanotic, conventional, epithelioid, neuroblastoma-like, and ancient [8]. Furthermore, PS is characterized by a multinodular (plexiform) growth pattern [9]. It most commonly occurs in the head, neck, or upper extremities, primarily in the skin or subcutaneous tissue [2,4]. It often presents as a slow-growing, asymptomatic lesion [1]. PS typically occurs in childhood and has equal male to female dominance [1].

Additionally, PS usually presents as a solitary lesion. However, it can also present as multiple lesions, particularly in association with syndromes such as neurofibromatosis type-2 and schwannomatosis, which are linked to genetic mutations in the NF2 gene and the tumor suppressor genes SMARCB1 and LZTR1, respectively [5,10,11]. Table 1 summarizes key features to differentiate sporadic PS from hereditary PS (Table 1).

**Table 1:** Key features to differentiate sporadic PS from hereditary PS PS: Plexiform schwannoma. References: [1,10,11].

Feature	Sporadic PS	Hereditary PS
Number of lesions	Usually solitary	Multiple lesions
Genetic mutations	Absent	NF2 gene mutations, SMARCB1/LZTR1 mutations
Family history	Negative	Positive for NF2, SMARCB1/LZTR1 mutations
Clinical presentation and location	Single localized mass. Trunk, head, neck, and upper extremity.	Localized or diffuse pain or asymptomatic mass. Neuropathy, tinnitus, hearing loss and balance dysfunction. Bilateral vestibular schwannomas, meningioma, spinal nerves and peripheral nerves schwannomas
Associated conditions	None	Neurofibromatosis or schwannomatosis syndromes
Risk of recurrence	Rare	Higher risk of recurrence
Risk of malignant transformation	Extremely rare	Rare

Differentiating plexiform schwannoma from other entities, specifically plexiform neurofibroma and malignant peripheral nerve sheath tumor, is crucial due to the differing risks of malignant transformation [1,3,12]. Both PS and plexiform neurofibroma tumors can look similar under a light microscope. However, the presence of Antoni A, which shows a nuclear palisading pattern and Verocay bodies and Antoni B, which shows a hypocellular myxoid pattern, are distinguishing characteristics [1,4,9]. Furthermore, S100 expression would be weakly positive in plexiform neurofibroma, while strongly positive in plexiform schwannoma [1,4,8,9]. Table 2 summarizes the histological, clinical, and immunohistochemical features of PS, neurofibroma, and malignant peripheral nerve sheath tumor (Table 2).

**Table 2:** Histological, clinical and immunohistochemical features of PS, neurofibroma and malignant peripheral nerve sheath tumor PS: Plexiform schwannoma; ±: positive or negative; +: positive; -: negative. References: [1,3,8,13,14].

Tumor	Histological and clinical features	Immunohistochemistry	Type
Plexiform schwannoma	Antoni A, Antoni B, Verocay bodies. Well-circumscribed, encapsulated, benign.	S100+, SOX10+, weak CD34+, EMA-.	Benign.
Neurofibroma	Hypocellular, myxoid areas without hypercellular areas. Lacks capsule, formed of spindle cells, shredded carrot collagen and mast cells.	CD34+, weak S100+, weak SOX10+, focal calretinin+, strong collagen type IV+.	Benign. Plexiform Neurofibroma has a risk of malignant transformation.
Malignant peripheral nerve sheath tumor	Hypocellular, pleomorphic nuclei, high mitotic activity, necrosis. Infiltrative growth.	Patchy S100±, patchy SOX10±, Ki67 high. Desmin+, myogenin+, myoD1+.	Malignant.

Plexiform schwannomas are typically benign peripheral nerve sheath tumors, and reports of malignant transformation are exceedingly rare [1]. PS lesions, if accessible, can be managed operatively by either incomplete or complete surgical resection, while inaccessible lesions can be managed by observation. Complications of Surgical excision include nerve damage [2,6,7]. Our patient falls into the age group of PS presentation, and his case was classical to several other cases in the literature with a single encapsulated lesion. However, unlike most cases, the location is rather unique and has only been reported a handful of times, including a case of a 33-year-old with a lesion that has been present since he was 13 years of age in the phalanx of the second toe of the right foot [15]. Similar to our patient, the histopathological findings of Araghi et al. also showed Antoni A and Antoni B areas with strong S100 positivity for Schwann cells [15]. This comparison displays the significance of considering plexiform schwannoma as a diagnosis, irrespective of age or location of the lesion, as it could conveniently rule out more malignant pathologies [3,16]. In terms of management, our patient’s treating team has favored complete surgical resection due to the lower risk of recurrence compared to incomplete resection [2,6,7]. The surgery was uneventful, with no nerve damage postoperatively. In the months after the surgery, the patient has shown no signs of recurrence and, to this day, remains free from further lesions.

## Conclusions

Despite being one of the rare subtypes of schwannomas, plexiform schwannomas are still a possible diagnosis, especially in young children. They could easily be mistaken for more sinister diagnoses, considering they share similar histopathology features. Thus, recognizing their characteristics and presentations is important to help exclude life-altering differentials. These schwannomas can easily be managed and diagnosed when coupled with the right clinical knowledge. Hence, physicians should be aware of these schwannomas.

## References

[1] Mortazavi N, Novin K, Zerehpoosh FB, Sadatsafavi M (2017). Plexiform schwannoma of the finger: a case report and literature review. Indian Dermatol Online J.

[2] Moini J, Samsam M, Avgeropoulos N (2025). Schwannoma. Epidemiology of Brain and Spinal Tumors.

[3] Rodriguez FJ, Folpe AL, Giannini C, Perry A (2012). Pathology of peripheral nerve sheath tumors: diagnostic overview and update on selected diagnostic problems. Acta Neuropathol.

[4] Ejiyooye TF, Dirisanala S, Makky Abouzied H, Mahjabeen SS, Sajjad T, Khan A (2022). A rare case of plexiform schwannoma of the little finger and its management: a case report. Cureus.

[5] Ko JY, Kim JE, Kim YH, Ro YS (2009). Cutaneous plexiform schwannomas in a patient with neurofibromatosis type 2. Ann Dermatol.

[6] Perry A, J. Brat D (2010). Peripheral Nerve Sheath Tumors. Practical Surgical Neuropathology: A Diagnostic Approach.

[7] Neville BW, Damm DD, Allen CM, Chi AC (2019). Soft Tissue Tumors. Color Atlas of Oral and Maxillofacial Diseases.

[8] (2025). Schwannoma - soft tissue. https://www.pathologyoutlines.com/topic/softtissueschwannoma.html.

[9] Berg JC, Scheithauer BW, Spinner RJ, Allen CM, Koutlas IG (2008). Plexiform schwannoma: a clinicopathologic overview with emphasis on the head and neck region. Hum Pathol.

[10] Dhamija R, Plotkin S, Gomes A, Babovic-Vuksanovic D (2018). LZTR1- and SMARCB1-Related Schwannomatosis. GeneReviews.

[11] Evans DG (2025). NF2-Related Schwannomatosis. GeneReviews.

[12] Lim HS, Jung J, Chung KY (2004). Neurofibromatosis type 2 with multiple plexiform schwannomas. Int J Dermatol.

[13] (2025). Neurofibroma - soft tissue. https://www.pathologyoutlines.com/topic/softtissueneurofibroma.html.

[14] (2025). Malignant peripheral nerve sheath tumor - soft tissue. https://www.pathologyoutlines.com/topic/softtissuempnst.html.

[15] Araghi F, Tabary M, Kamyab K, Forouzanfar MM, Robati RM (2021). A rare case of plexiform schwannoma on the foot. Clin Case Rep.

[16] Santos PP, Freitas VS, Pinto LP, Freitas Rde A, de Souza LB (2010). Clinicopathologic analysis of 7 cases of oral schwannoma and review of the literature. Ann Diagn Pathol.

